# Clinical assessment of recurrent cancer: a Danish cohort study in general practice

**DOI:** 10.3399/BJGP.2025.0316

**Published:** 2026-01-13

**Authors:** Kasper Grooss, Linda Aagaard Rasmussen, Kaj Sparle Christensen, Anette Fischer Pedersen, Alina Zalounina Falborg, Peter Vedsted

**Affiliations:** 1 Research Unit for General Practice, Aarhus, Denmark; 2 Department of Public Health, Aarhus University, Aarhus, Denmark; 3 Department of Clinical Medicine, Aarhus University, Aarhus, Denmark; 4 Center for General Practice, Aalborg University, Aalborg, Denmark; 5 University Clinic for Innovative Patient Pathways, Department of Clinical Medicine, Aarhus University, Aarhus, Denmark

**Keywords:** cancer, cohort studies, Denmark, general practice, neoplasm recurrence, local, referral and consultation, surveys and questionnaires

## Abstract

**Background:**

GPs may detect cancer recurrence (CR) between specialised follow-up visits or after completed follow-up. Knowledge of CR detection is needed to inform GP decision making in general practice.

**Aim:**

To examine how often GPs suspected cancer in patients presenting with CR, diagnostic actions taken, and how cancer suspicion and diagnostic actions were associated with the time to recurrence diagnosis.

**Design and setting:**

A retrospective cohort study was conducted linking survey data and national register data.

**Method:**

Patients diagnosed with recurrence (of one of seven cancer types) between 1 January 2022 and 31 May 2024 were included. Their GPs provided details on the diagnostic process.

**Results:**

The GP survey response rate was 48% (1265/2611), and 469 patients presented with signs or symptoms of recurrence. The GPs suspected cancer at the first consultation in 226 patients (48%). A referral was made for diagnostic evaluation in 282 (60%) patients, with fast-track cancer pathways being the most frequent (48%) and fastest referral mode. Diagnostic intervals differed across cancer types at the 90th percentile. The longest intervals were seen for melanoma recurrence, which was 47 days longer than colorectal CR. The median diagnostic interval was 60 days shorter when GPs suspected cancer compared with no suspicion of cancer or other serious disease.

**Conclusion:**

The GPs suspected cancer in approximately half of the patients and referred three in five presenting with signs or symptoms of recurrence. In approximately one-third, the GP had no suspicion. The length of the diagnostic interval differed considerably between the seven investigated cancer types.

## How this fits in

As more patients receive curative cancer treatment, the number of cancer recurrences increases. GPs are crucial for timely recurrence detection, as symptoms often emerge between scheduled follow-up visits or after completed follow-up. GPs suspected cancer in approximately half of patients consulting for symptoms of recurrence, and three in five were referred for diagnostic evaluation. Cancer suspicion was linked with the greatest reduction in median diagnostic interval.

## Introduction

Eight per cent of the Danish population lives with a cancer diagnosis, with the number of cancer survivors growing by 4% annually.^1^ Enhancing the capacity and quality of care for cancer survivors is increasingly important. Although no evidence has yet linked early detection of cancer recurrence (CR) directly to improved survival, early detection of CR remains a central part of cancer survivor care.^2^ As treatment options have advanced significantly in recent years, a timely diagnosis may improve the prognosis for patients with CR.^3–5^ Cancer survivors often consult their GP,^6^ and the frequency of visits tends to increase before a CR diagnosis.^7^ Thus, the GP may play a key role in early detection of CR.^8–10^

CR symptoms often differ from primary cancer symptoms, particularly for distant recurrence.^11^ Many CR symptoms are common in the general population.^12,13^ Additionally, comorbidities^14^ and late effects of the primary cancer and its treatment^15^ may further complicate the clinical picture.

Therefore, deciding which patients require diagnostic evaluation and when to act can be difficult. Fast-track cancer patient pathways are tailored for primary cancer symptoms and risk profiles,^16^ making them less applicable to patients with CR, who may not meet referral criteria. These challenges may contribute to longer diagnostic intervals in general practice.

The GPs’ clinical assessments and diagnostic actions for cancer survivors should expedite the diagnostic process for patients with CR,^9,17^ while balancing potential harms and costs.^18^ Evidence on CR detection in general practice is limited. Therefore, to inform clinical guidelines, there needs to be an understanding of GP decision making when cancer survivors present with symptoms.

This study aimed to estimate the frequency of cancer suspicion among GPs and their diagnostic actions when patients with CR of seven cancer types first presented with signs or symptoms in general practice. Further, the study aimed to analyse how GP cancer suspicion is associated with diagnostic actions and length of diagnostic interval.

## Method

### Design

A retrospective national cohort study was conducted, linking GP survey data with individual-level register data.

### Setting

Denmark offers free (tax-funded) health care, and 99% of residents are registered with a general practice, which they must contact for medical advice. GPs serve as gatekeepers to specialised health care and may refer patients to hospitals or clinics, thereby transferring the responsibility for diagnostic evaluation. GPs keep detailed medical records, including hospital discharge letters, outpatient clinic notes, and test results.^19^ After cancer treatment, patients enter a specialised follow-up programme with CR surveillance and management of late effects.

### Participants

Patients were sampled with a first-time diagnosis of primary colorectal, lung, melanoma, breast, endometrial, ovarian, or bladder cancer (International Classification of Diseases, Tenth Revision [ICD-10]: C18–20, C34, C43, C50, C54, C56, C67) in 2012–2024 recorded in the Danish Cancer Register^20^ and the Danish Colorectal Cancer Group Database^21^ for colorectal cancer. The Danish National Patient Register (DNPR)^22^ was used to sample the most recently diagnosed patients. Validated register-based algorithms were applied to identify CR cases between January 2022 and May 2024.^23–28^ CR was defined as the return of cancer at the original site or a distant location after curative treatment and a subsequent remission period with no register-based evidence of ongoing disease. This period was defined separately for each cancer type: 90–180 days after surgery or 30–90 days after the last day of oncological treatment, whichever came last.^23–28^ CR indicators included diagnosis and procedure codes from the DNPR and pathology test results from the Danish National Pathology Register.^29^

### Data collection

Survey data were collected from January 2023 until July 2024. Each CR patient’s general practice received an invitation. Initially, patients diagnosed with CR within 12 months were targeted. Subsequently, GPs of patients with newly identified CR were invited every fourth month. Responders were remunerated with DKK 146 (EUR 20; approximately 17.50 GBP). Non-responders received a reminder after 3 weeks.

### Survey data

Each GP was asked to indicate, based on their current knowledge and the patient’s medical record, an index consultation, defined as the first date of presenting with CR signs or symptoms in general practice. If such a date was identified, the GP provided information on the following three variables:

1. Overall assessment when the patient first presented with signs or symptoms in general practice (suspected cancer, suspected other serious disease, did not suspect cancer or other serious disease).
2. Activities initiated before the recurrence diagnosis (watchful waiting, awaited cancer follow-up visit, local tests, confer with specialist via phone or letter, referral for diagnostic evaluation); referral was further categorised (cancer patient pathway, cancer responsible hospital department, specialist in private practice or outpatient clinic, diagnostic imaging, none of the above).
3. Follow-up status, that is, enrolled in active, specialised follow-up when the recurrence was diagnosed (yes, no, do not know).

### Diagnostic intervals

Diagnostic intervals were calculated as the time between index consultation and CR diagnosis.^20^ Interval length was truncated at 365 days (*n* = 11). Negative values of up to –100 days were adjusted to 0 days (*n* = 27), as they were likely owing to inaccurate CR diagnosis dates. If a negative interval length was longer than –100 days (*n* = 38), or the interval length exceeded 365 days and the GP reported ‘no delay’ (*n* = 15), the interval was coded as ‘missing’, as it was likely caused by an erroneous GP-specified date.

### Other variables and data sources

Comorbidity was defined according to the Charlson Comorbidity Index (CCI),^30^ based on diagnosis codes registered in the DNPR from hospital contacts in the 10 years preceding the CR diagnosis date, excluding cancer-related diagnoses. CCI scores were categorised into ‘low’ (score 0), ‘medium’ (scores 1–2), and ‘high’ (scores >2).

Statistics Denmark^31^ provided information on sex, age at CR diagnosis, educational level, and cohabitation status. Educational level was categorised according to the International Standard Classification of Education (ISCED)^23^ into ‘low’ (<10 years), ‘medium’ (10–15 years), and ‘high’ (>15 years). Cohabitation status was categorised as ‘cohabitating’ (married or registered with a partner) or ‘living alone’ (widowed, divorced, or unmarried).

### Statistical methods

The GP’s assessment at index consultation and the diagnostic actions were categorised by cancer type, and the total number and percentage were presented for each category. Descriptive analyses were performed using the χ^2^ test.

Poisson regression with robust variance estimation was used to estimate risk ratios (RRs) with 95% confidence intervals (CIs) of the GP taking a specific diagnostic action as a function of GP assessment.

Quantile regression^32^ was used to estimate diagnostic intervals in calendar days at the 50th and 90th percentiles. Diagnostic intervals were derived by counting days using the ‘qcount’ procedure.^33,34^ Diagnostic interval differences were calculated as marginal effects.

A directed acyclic graph (DAG) was used to identify confounders for inclusion in the adjusted analyses (Supplementary Figure S1).^35^ Analyses were restricted to patients with complete data on all required variables. All analyses were performed in Stata (version 18.0).

## Results

### Participants

**Figure 1.**
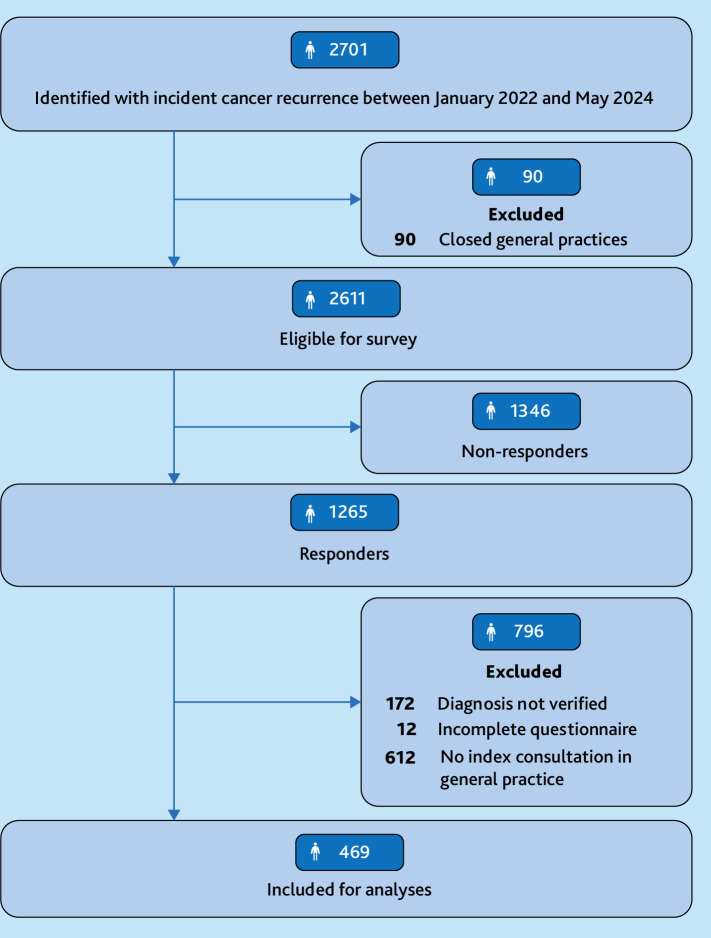
Study population flowchart.

In total, 469 patients with CR were included who had an index consultation in general practice (Figure 1). Patient characteristics were similar between patients of responding and non-responding GPs. Compared with patients with an index consultation in general practice, those without were more frequently in active follow-up (85% versus 50%), had higher rates of colorectal (28% versus 20%) and ovarian cancer (4% versus 1%), and lower rates of breast cancer (31% versus 43%). Table 1 presents the study population characteristics. Breast cancer was the most common type (43%). The proportion of patients in active follow-up at CR diagnosis ranged from 45%–50% for colorectal, breast, melanoma, and endometrial cancer to 71%–77% for lung, bladder, and ovarian cancer. The median time from primary cancer diagnosis to CR was 3.6 years (interquartile interval [IQI]: 1.5–7.1), and the median diagnostic interval for the CR was 49 days (IQI: 20–121).

**Table 1.** Patient characteristics, cancer type, and follow-up status for patients with cancer recurrence (January 2022 to May 2024) and an index consultation in general practice (*n* = 469)

	*n*	(%)
**Total**	469	(100.0)
**Sex**		
Male	146	(31.1)
Female	323	(68.9)
**Age group** (years)		
18–54	65	(13.9)
55–64	91	(19.4)
65–74	138	(29.4)
≥75	175	(37.3)
**Educational level^a^**		
Low	136	(29.0)
Medium	215	(45.8)
High	118	(25.2)
**Cohabitation status**		
Cohabitating	211	(45.0)
Living alone	258	(55.0)
**Comorbidity level^b^**		
Low	340	(72.5)
Medium	108	(23.0)
High	21	(4.5)
**Cancer type**		
Breast	204	(43.5)
Colorectal	96	(20.5)
Lung	69	(14.7)
Melanoma	52	(11.1)
Bladder	22	(4.7)
Endometrial	19	(4.1)
Ovarian	7	(1.5)
**Follow-up status**		
Active	235	(50.1)
Ended	213	(45.4)
Unknown	21	(4.5)

^a^According to the International Standard Classification of Education (ISCED),^28^ educational level was divided into low (<10 years), medium (10–15 years), and high (>15 years). ^b^According to the Charlson Comorbidity Index score, comorbidity level was calculated based on diagnosis codes registered in the Danish National Patient Register in the 10 years preceding a recurrence diagnosis (excluding cancer-related diagnoses), and scores were divided into low (0), medium (1–2), and high (≥3).

### Assessment at index consultation

**Figure 2.**
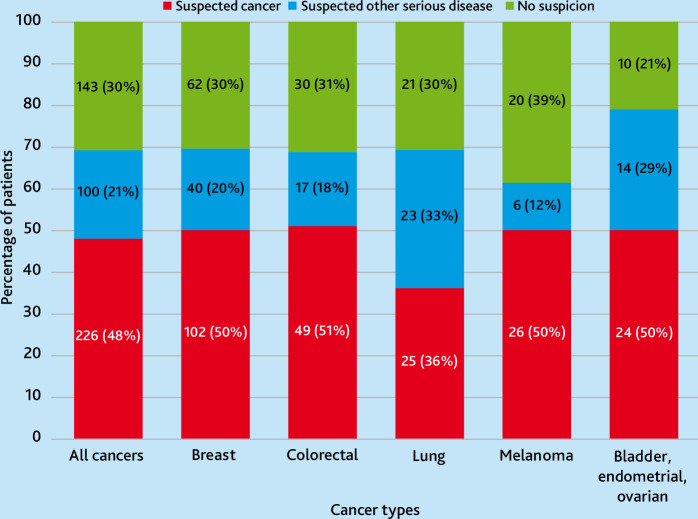
GP assessment at index consultation.

The GP suspected cancer in 226 (48%) patients, other serious disease in 100 (21%) patients, and neither cancer nor serious disease in 143 (30%) patients. Assessments were similar across cancer types, except for lung cancer (Figure 2,2), where GPs were less likely to suspect cancer (*P* = 0.031) and more likely to suspect other serious disease (*P* = 0.008) compared with the other six cancer types.

### Diagnostic actions

**Figure 3.**
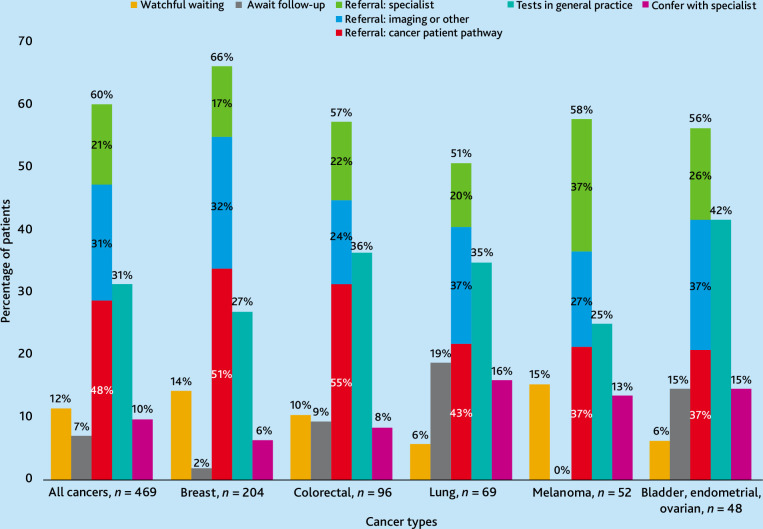
GP actions preceding cancer recurrence diagnosis.

Figure 3,3 illustrates diagnostic actions by cancer type. Referral for diagnostic evaluation was the most common diagnostic action for all cancer types (282/469, 60%), with cancer patient pathways representing the most frequently used referral modality (136/282, 48%). Notably, patients with melanoma were as likely to be referred to a specialist as to a cancer patient pathway.

For patients diagnosed with CR during active follow-up (*n* = 235), the GP awaited follow-up for 33 (14%), with the highest rates for patients with bladder, endometrial, or ovarian cancer (7/23, 30%), and lung cancer (13/48, 27%).

Table 2 shows the association between GP assessment at the index consultation and diagnostic actions. When neither cancer nor serious disease was suspected, the GPs were less likely to confer with a specialist (RR = 0.2, 95% CI = 0.1 to 0.5) and more likely to perform local tests (RR = 1.7, 95% CI = 1.2 to 2.4) compared with when cancer was suspected.

**Table 2.** Adjusted^,a,b^ risk ratios (RR) of GP actions (Part A) and referral modalities (Part B) by assessment at index consultation in general practice for patients with cancer recurrence (January 2022 to May 2024) (*n* = 469)

A: Actions by GP																	
		Watchful waiting				Await follow-up^c^			Tests in general practice^d^			Confer with specialist^e^			Referral for diagnostic evaluation		
	*n*	*n*	RR		CI 95%	*n*	RR	CI 95%	*n*	RR	CI 95%	*n*	RR	CI 95%	*n*	RR	CI 95%
Total	469	54				33			147			46			282		
**Assessment**																	
Suspected cancer	226	9	ref			11	ref		49	ref		33	ref		163	ref	
Suspected other serious disease	100	4	1.0		(0.3 to 3.2)	8	1.1	(0.4 to 2.7)	43	**2.0**	**(1.4 to 2.8)**	9	0.6	(0.3 to 1.2)	62	0.9	(0.7 to 1.0)
No suspicion	143	41	**6.9**		**(3.5 to 13.7)**	14	1.6	(0.8 to 3.4)	55	**1.7**	**(1.2 to 2.4)**	4	**0.2**	**(0.1 to 0.5)**	57	**0.6**	**(0.5 to 0.7)**
**B: Referral modalities**																	
		Cancer patient pathway				Specialist			Imaging or other								
	*n*	*n*	RR	CI 95%		*n*	RR	CI 95%	*n*	RR	CI 95%						
Total	282	136				60			87								
**Assessment**																	
Suspected cancer	163	117	ref			22	ref		25	ref							
Suspected other serious disease	62	13	**0.3**	**(0.2 to 0.5)**		20	**2.0**	**(1.1 to 3.6)**	29	**2.4**	**(1.5 to 3.8)**						
No suspicion	57	6	**0.1**	**(0.0 to 0.2)**		18	1.3	(0.7 to 2.4)	33	**2.0**	**(1.3 to 3.3)**						

^a^Crude estimates were similar to adjusted estimates and, therefore, omitted from the table. ^b^Adjusted for age, sex, educational level, follow-up status, and comorbidity level. ^c^Among patients in active follow-up (*n *= 235).^d^For example, blood tests, spirometry, urinalysis. ^e^Non-referral contact by phone or letter to a specialist. Bold indicates statistical significance, *P*≤0.05. RR = risk ratio.

### Diagnostic intervals

When the GP suspected cancer, the interval was 60 days shorter (95% CI = 51 to 69) at the 50th percentile compared with when the GP suspected neither cancer nor serious disease. No differences were observed at the 90th percentile (Table 3).

**Table 3.** Differences in length of diagnostic interval in calendar days at the 50th and 90th percentiles^a^ for patients with an index consultation in general practice. Patients with missing diagnostic interval were omitted (*n* = 53) (*n* = 416)

			Crude		Adjusted^b^			Crude			Adjusted^b^		
	*n*	50th	Diff	(CI 95%)	50th	Diff	(CI 95%)	90^th^	Diff	(CI 95%)	90th	Diff	(CI 95%)
**Index consultation assessment**													
No suspicion	127	83	ref		92	ref		230	ref		151	ref	
Suspected cancer	197	31	**–52**	(**–66 to –38**)	32	**–60**	(**–69 to –51**)	218	**–12**	(**–22 to –1**)	151	0	(–11 to 10)
Suspected other serious disease	92	36	**–47**	(**–53 to –41**)	42	**–50**	(**–66 to –34**)	199	**–31**	(**–39 to –22**)	116	**–35**	(**–39 to –31**)
**Cancer types**													
Colorectal	82	54	ref		53	ref		238	ref		234	ref	
Breast	193	45	**–9**	(**–17 to –1**)	43	–10	(–36 to 15)	209	**–29**	(**–39 to –19**)	219	–15	(–30 to 0)
Lung	54	52	–2	(–10 to 7)	53	0	(–13 to 14)	185	**–53**	(**–59 to –46**)	174	**–60**	(**–78 to –43**)
Melanoma	46	49	–5	(–10 to 0)	47	–6	(–24 to 12)	347	**109**	(**102 to 115**)	281	**47**	(**32 to 62**)
Bladder	19	30	**–24**	(**–30 to –17**)	31	**–22**	(**–41 to –4**)	175	**–63**	(**–68 to –57**)	137	**–97**	(**–112 to –82**)
Endometrial	16	44	**–10**	(**–15 to –5**)	44	–9	(–21 to 2)	155	**–83**	(**–88 to –77**)	153	**–81**	(**–97 to –64**)
Ovarian	6	68	**14**	(**9 to 19**)	69	16	(–11 to 43)	173	**–65**	(**–70 to –60**)	237	3	(–18 to 23)
**GP actions^c^**													
Watchful waiting	49	100	**56**	(**44 to 67**)	74	**37**	(**32 to 43**)	283	**72**	(**60 to 84**)	226	**25**	(**15 to 35**)
Await follow-up^d^	29	78	**26**	(**19 to 32**)	62	**20**	(**6 to 33**)	185	**–14**	(**–23 to –6**)	98	**–20**	(**–25 to –14**)
Tests in general practice^e^	128	53	7	(–7 to 20)	52	10	(–1 to 21)	214	–1	(–12 to 11)	169	**25**	(**19 to 30**)
Confer with specialist^f^	41	36	**–13**	(**–17 to –9**)	37	–8	(–21 to 5)	140	**–77**	(**–89 to –65**)	134	**–61**	(**–75 to –47**)
Referral	255	14	**–24**	(**–34 to –14**)	14	**–16**	(**–27 to –5**)	201	–8	(–20 to 4)	126	**–18**	(**–24 to –12**)
**Referral modality**													
Cancer patient pathway	123	23	ref		19	ref		181	ref		204	ref	
Specialist	54	64	**41**	(**33 to 49**)	52	33	(–22 to 88)	209	**28**	(**21 to 36**)	243	**39**	(**3 to 76**)
Imaging or other	78	54	**31**	(**27 to 36**)	55	**36**	(**13 to 58**)	279	**98**	(**89 to 106**)	231	**27**	(**20 to 34**)

^a^Pseudo-centiles were used for reporting of 50th and 90th percentile diagnostic interval length of references; for non-references the total diagnostic interval lengths are calculated from adding the point estimate to the reference value. ^b^Index consultation assessment adjusted for sex, age, follow-up status, comorbidity, and educational level. Cancer types adjusted for age and educational level. GP actions and referral modality were adjusted for age, sex, comorbidity level, follow-up status, and educational level. ^c^Separate analyses were conducted for each action with the reference group comprising all patients without that particular action (*n* = 416). ^d^Among patients in active follow-up (*n *= 235). ^e^Such as blood tests, urinalysis, spirometry. ^f^Non-referral contact by phone or letter to specialist. Bold indicates statistical significance, *P*≤0.05. Diff = difference.

Intervals at the 90th percentile varied across cancer types. Compared with patients with colorectal cancer, patients with melanoma had intervals that were 47 days longer (95% CI = 32 to 62), while patients with bladder cancer had intervals 97 days shorter (95% CI = 82 to 112).

For diagnostic actions, the interval was 37 days longer (95% CI = 32 to 43) at the 50th percentile if the GP applied watchful waiting. The interval was 61 days shorter (95% CI = 47 to 75) at the 90th percentile if the GP conferred with a specialist. Notably, performing tests in general practice prolonged the interval by 10 days (95% CI = -1 to 21) at the 50th percentile and by 25 days (95% CI = 19 to 30) at the 90th percentile. Referral to a cancer patient pathway was associated with shorter intervals.

## Discussion

### Summary

When patients with CR first consulted their GP, cancer was suspected in approximately half of the patients, and three in five were referred to diagnostic evaluation. Cancer patient pathways were the most frequent referral modality and were associated with the shortest diagnostic intervals. Cancer suspicion was associated with diagnostic actions and a 2-month shorter median diagnostic interval compared with when neither cancer nor serious disease was suspected.

### Strengths and limitations

Identifying patients with CR is a major challenge in CR research, as CRs are not routinely registered. Using register-based algorithms, the high validity of Danish health registers were leveraged to identify a national cohort of patients with CR.

Surveying GPs offered key advantages. It mitigated selection bias from patient health and socioeconomic status, allowed inclusion of deceased patients, and limited recall bias by leveraging the GPs’ access to detailed medical records.

The study design is susceptible to selection and information bias. Although GPs verified CR diagnoses, thereby reducing false positives, the algorithms may have missed patients with CR. Reported sensitivities ranged from 83% to 97%.^23–28^ The most comorbid and frail patients were more likely to be missed, as their health conditions may preclude biopsy or cancer treatment, which may limit the detectable indicators of CR.^23–28^ Not including these patients may have led to shorter diagnostic intervals, as neither they nor the GPs may have prioritised rapid diagnosis. Including them, however, could reduce the clinical relevance of these findings.

Although the GP response rate exceeded those in recent comparable surveys in Danish general practice,^36,37^ the 48% was lower than desired and reduced statistical precision. Characteristics were similar between patients of responding and non-responding GPs, but non-response bias cannot be ruled out. Some GPs may have avoided responding to conceal delays, which might have underestimated the diagnostic intervals. However, GP anonymity and the potential motivation from adverse outcomes are likely to have mitigated this factor.

The questionnaire date-picker defaulted to the current date. When GPs did not adjust appropriately, it led to intervals either 1 year shorter (*n* = 38) or 1 year longer (*n* = 15) than intended. To conserve the accuracy of interval data, these 53 entries were set to missing. As such errors were likely random, they are unlikely to have biased the results.

While recall bias could have influenced GP reports of their initial assessment, especially when outcomes were favourable, the lack of diagnostic interval differences at the 90th percentile between GPs suspecting cancer and those suspecting neither cancer nor serious disease indicates that such bias was minimal.

### Comparison with existing literature

To the authors’ knowledge, no studies have examined GP assessments, diagnostic actions, or diagnostic intervals specifically for patients with CR presenting in general practice. While similar studies exist for primary cancer diagnosis, direct comparisons should consider that cancer survivors fear CR^38^ and may be more inclined to seek diagnostic evaluation.

Previous studies have reported similar rates of alarm symptoms in patients presenting with primary cancer as the rate of cancer suspicion in this study.^39–42^ Notably, the lower rate of cancer suspicion in patients with recurrent lung cancer mirrors the lower rate of alarm symptoms in patients with primary lung cancer.^39^ This may be owing to the high prevalence of chronic lung diseases in patients with lung cancer,^43^ making it difficult to distinguish between chronic and cancer-related symptoms. Still, the authors expected a previous cancer to prompt higher suspicion among GPs.

Referral rates to fast-track cancer pathways in patients with melanoma, colorectal, and lung cancer were similar to those seen for primary cancer.^39^ However, patients with recurrent breast cancer had lower rates than those seen in primary care (51% versus 63%), which might be explained by a high proportion of distant recurrences and the typically palpable presentation of primary tumours.^11^

### Implications for practice

Cancer survivors have an elevated risk of developing CR.^44^ Nevertheless, the GPs did not suspect cancer more often in cancer survivors than in patients with primary cancer, and they did not refer survivors more often to cancer patient pathways. This highlights the difficult task of interpreting signs and symptoms in cancer survivors. These findings suggest a need for increased clinical focus on symptoms and signs, and relevant patient pathways for those at risk of CR. More clinical research on the CR diagnosis in general practice seems relevant.

Performing local tests in general practice was a frequent diagnostic action, but this approach may be problematic for patients at risk of CR. While local tests may (or may not) help confirm cancer suspicion, potential benefits may be outweighed by a longer diagnostic interval, which at the 90th percentile was similar to the interval increase for performing watchful waiting.

The considerable variation in diagnostic intervals at the 90th percentile across cancer types may reflect differences in the proportion of patients presenting with atypical symptoms, as well as the rate at which these symptoms progress. These findings suggest that the current approach for CR detection in general practice may not effectively serve patients of all cancer types.

Notably, the most substantial reduction in median diagnostic intervals was observed when the GPs suspected cancer. This suggests a need for tailored guidelines on presentation patterns to increase GP awareness of potential CRs.

Patients with CR of melanoma were most likely to experience the longest diagnostic intervals. This may reflect the high rates of distant CR, which often present with non-specific symptoms.^45^ Such presentations likely contributed to the high rates of watchful waiting owing to low cancer suspicion. Adopting a more proactive diagnostic approach, with lower referral thresholds, could help reduce diagnostic intervals for these patients.

A systematic, tailored approach to CR detection in general practice is needed. Our findings provide insights that can inform and support the clinical development. We also need research to assess when an earlier detection of CR may improve patient outcomes.

## Data

The technical appendix and statistical codes are available from the corresponding author. Because of Danish data protection legislation, the dataset is accessible only to pre-approved collaborative partners.
